# Systemic lupus erythematosus combined with Castleman disease and secondary paraneoplastic pemphigus: a case report

**DOI:** 10.1186/s12969-023-00871-2

**Published:** 2023-10-19

**Authors:** Xin Ma, Jiyuan Li, Linlin Fan, Hongwei Jiang, Gaishao Shi, Dongfeng Ge, Xiaofei Shi

**Affiliations:** 1 Department of Rheumatology and Immunology, The First Affiliated Hospital, and College of Clinical Medicine of Henan University of Science and Technology, No,24 Jinghua Road, Luoyang, China; 2 Department of Thoracic Surgery, The First Affiliated Hospital, and College of Clinicalcal Medicine of Henan University of Science and Technology, No,24 Jinghua Road, Luoyang, China; 3 Henan Key Laboratory of Rare Diseases, Endocrinology and Metabolism Center, The First Affiliated Hospital, and College of Clinical Medicine of Henan University of Science and Technology, No,24 Jinghua Road, Luoyang, China; 4 Department of Pathology, The First Affiliated Hospital, and College of Clinical Medicine of Henan University of Science and Technology, No,24 Jinghua Road, Luoyang, China

**Keywords:** Systemic lupus erythematosus, Castleman disease, Pemphigus, Paraneoplastic syndromes

## Abstract

**Background:**

The literature describes a case of systemic lupus erythematosus (SLE) complicated with Castleman’s disease (CD) and secondary paraneoplastic pemphigus (PNP).

**Case presentation:**

A 12-year-old female presented with a neck mass, rash, arthralgia, and skin and mouth ulceration for 5 years were admitted. All blood cells were low. Multiple autoantibodies associated with SLE were positive. The pathology of the neck mass revealed the classical manifestations of CD. She was treated with prednisone, hydroxychloroquine, leflunomide, thalidomide, and dressings. Pathological examination of the skin revealed PNP. The neck mass was removed and continued to take antirheumatic drugs. At subsequent follow-up, the patient’s disease status was stable and the skin mucosal lesion did not recur.

**Conclusion:**

The case of simultaneous SLE, CD, and PNP in children was rarely reported, and the correct diagnosis of the disease will help to take timely treatment.

## Background

Systemic lupus erythematosus (SLE) is a multisystem autoimmune disorder of the connective tissues characterized by autoantibodies that target nuclear antigens, remissions and flares, and a highly variable clinical presentation, disease course, and prognosis [1–5]. Multiple organs and systems may be involved in SLE, including the kidneys, skin, musculoskeletal system, cardiovascular system, central and peripheral nervous systems, and blood [1–5]. SLE is more common in women, with a female-to-male ratio of 2–5:1 in children [4, 5]. The etiology of SLE is unknown, but likely involves loss of self-tolerance and resulting autoimmunity in individuals with genetic predisposition after exposure to environmental triggers in the setting of various immunologic and hormonal factors [3–5].

Castleman disease (CD) is an uncommon group of heterogeneous lymphoproliferative disorders that cause nonmalignant lymphadenopathy related to increased release of cytokines, particularly interleukin-6 (IL-6) [6–9]. In the United States, the incidence of unicentric CD is estimated to be between 15 and 19 cases per million patient-years, and the incidence of multicentric CD is estimated to be between 5 and 6 per million patient-years [9].

Pemphigus is a rare (incidence of 0.75-5 per million people-year) autoimmune disease characterized by chronic, non-healing, painful mouth ulcers and progressive blistering, bullae, and erosions of the skin [10–12]. The skin lesions are commonly found on the face, scalp, and torso [10–12]. Mucosal lesions may also be found on the conjunctiva, esophagus, or genitalia [10–12]. Without treatment, the prognosis is poor, with mortality at about 75% at 1 year [10]. With treatment, mortality is reduced to 10%[10, 13]. The underlying trigger is usually unknown [11], but paraneoplastic pemphigus (PNP) can be associated with CD or be present independently from CD [6, 7]. Paraneoplastic pemphigus is a common autoimmune syndrome of lymphoma, leukemia, CD and other malignant tumors [14]. The main manifestations are oral ulcers and skin lesions. Immunosuppression is the main treatment method [14].

Localized lymphadenopathy can be observed in patients with SLE, and biopsies to exclude malignancy can reveal a CD morphology [15, 16]. Multicentric CD might be closely associated with autoimmune diseases [17, 18]. Cases of CD mimicking SLE have also been reported [19, 20]. However, cases of PNP combined with SLE have been rare reported in children [21, 22].

Here, we report a rare case of SLE combined with CD and PNP.

## Case presentation

A 12-year-old female presented with rash, arthralgia, and skin ulceration for 5 years and was admitted to the First Affiliated Hospital of Henan University of Science and Technology on December 13, 2015. In 2010, she had multiple rashes and ulcers on the trunk and limbs, accompanied by exudation of a dark red liquid. She also had ulceration, peeling, and scabbing on the tongue tip and lips, affecting food intake. Her interphalangeal joints of both hands were swollen due to synovitis. At another hospital, glucocorticoid treatment was given, and the skin lesions were improved. In 2011, the rash worsened again. The fingers of both hands were swollen, with mild flexion and deformity. She tested positive for the antinuclear antibody (ANA) and anti-double-stranded DNA (dsDNA) antibody at another hospital. She received prednisone 30 mg/day and hydroxychloroquine sulfate 200 mg/day. After 1 month of treatment, most skin lesions were improved, but the lesions in the oral cavity and on hands and feet were not. She intermittently took prednisone 5-20 mg/day orally, but her parents did not take her to the hospital for medical treatment until 2015.

**Fig. 1:**
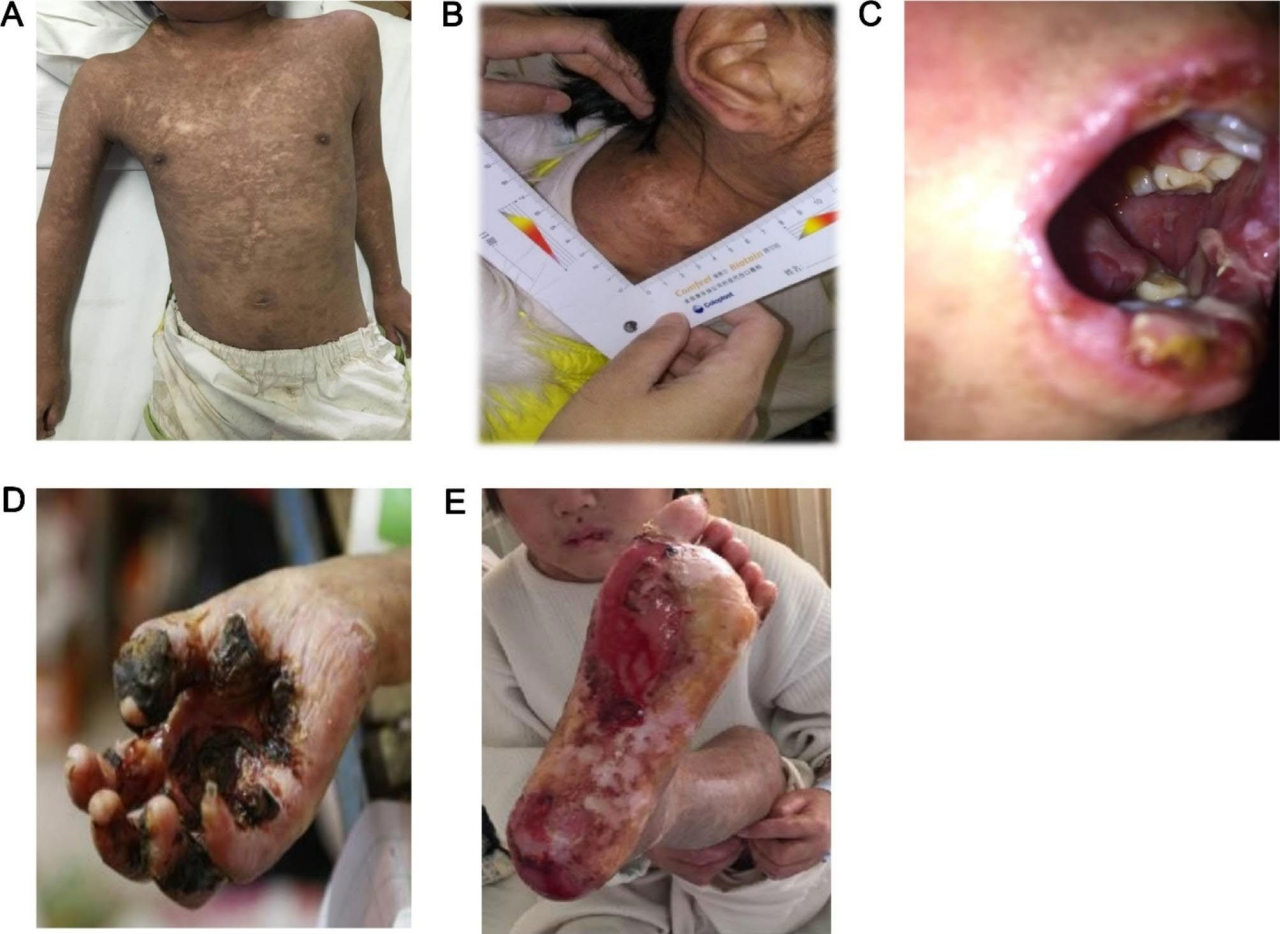
Signs and symptoms at presentation of a 12-year-old girl with systemic lupus erythematosus, Castleman disease, and paraneoplastic pemphigus. (**A**) Old brown pigmentation over the whole body. (**B**) Right neck mass. (**C**) Multiple ulcers and erosions on the mucous membranes of tongue tip and lips, and red scabs on the lips. (**D**) Finger flexion and deformity, and ulcerations and scabs of the skin on the palm. (**E**) Bilateral plantar areas

At admission in 2015, her medical history included the presence of a mass on the right neck since childhood that enlarged with age. It was once diagnosed with lymphadenectasis, but no treatment was given because of no discomfort. She lost a thumb tip in 2011 due to trauma. She was a first child, natural delivery, with a birth weight of 2.6 kg. Growth and development were normal. Her mother had a history of mental disorders, and the father had a mild intellectual disability. She had a younger brother, who was healthy. She had no history of genetic diseases and no familial history of such signs and symptoms. The patient was malnourished and was emaciated with a height of 110 cm and weight of 18 kg. Her skin was very thin, with old brown pigmentation over the whole body (Fig. 1A). A mass was present on the right neck, measuring about 6 × 7 cm (Fig. 1B). The superficial lymph nodes were not enlarged. Multiple ulcers and erosions were noted on the mucous membranes of tongue tip and lips, and red scabs were seen on the lips (Fig. 1C). Finger flexion and deformity were seen, and the skin on the palm was ulcerated and scabbed (Fig. 1D). The skin of bilateral plantar areas was ulcerated with bleeding and a small amount of brown secretions (Fig. 1E).

Blood routine examination revealed low white blood cells (3.14 × 10^9^/L), low lymphocytes (1.4 × 10^9^/L), low red blood cells (3.84 × 10^12^/L), low hemoglobin (64.2 g/L), low hematocrit (25.9%), low mean corpuscular volume (67.5 fl.), low mean corpuscular hemoglobin (19.6 pg), low platelets (71 × 10^9^/L), high erythrocyte sedimentation rate (94 mm/h), high C-reactive protein (25.2 mg/L), albumin at 32 g/L, and high ferritin (685 ng/ml). ANA, anti-ds-DNA, anti-Ro, anti-nucleosome, and anticardiolipin antibodies were positive, while anti-CCP and anti-RF were negative. IgG was elevated (28.6/L), IgM was normal, C3 was low (0.43 g/L), and C4 was low (0.17 g/L). The bone marrow puncture was normal. The secretion culture showed no bacterial growth. Echocardiography showed tachycardia, and there was a small amount of pericardial effusion. The neck mass was solid and was considered as an enlarged lymph node. There were multiple unequal-sized hypoechoic masses, and abundant blood flow signals were seen in the peripheral region and inside. Hand X-ray showed osteoporosis of both hands, flexion of the distal part of the ring finger of the right hand, and unclear bone structure. Chest CT revealed a small amount of exudation in both lungs and small pericardial effusions. Abdominal CT showed that the spleen was slightly enlarged and revealed a stone in the vermiform appendix.

**Fig. 2:**
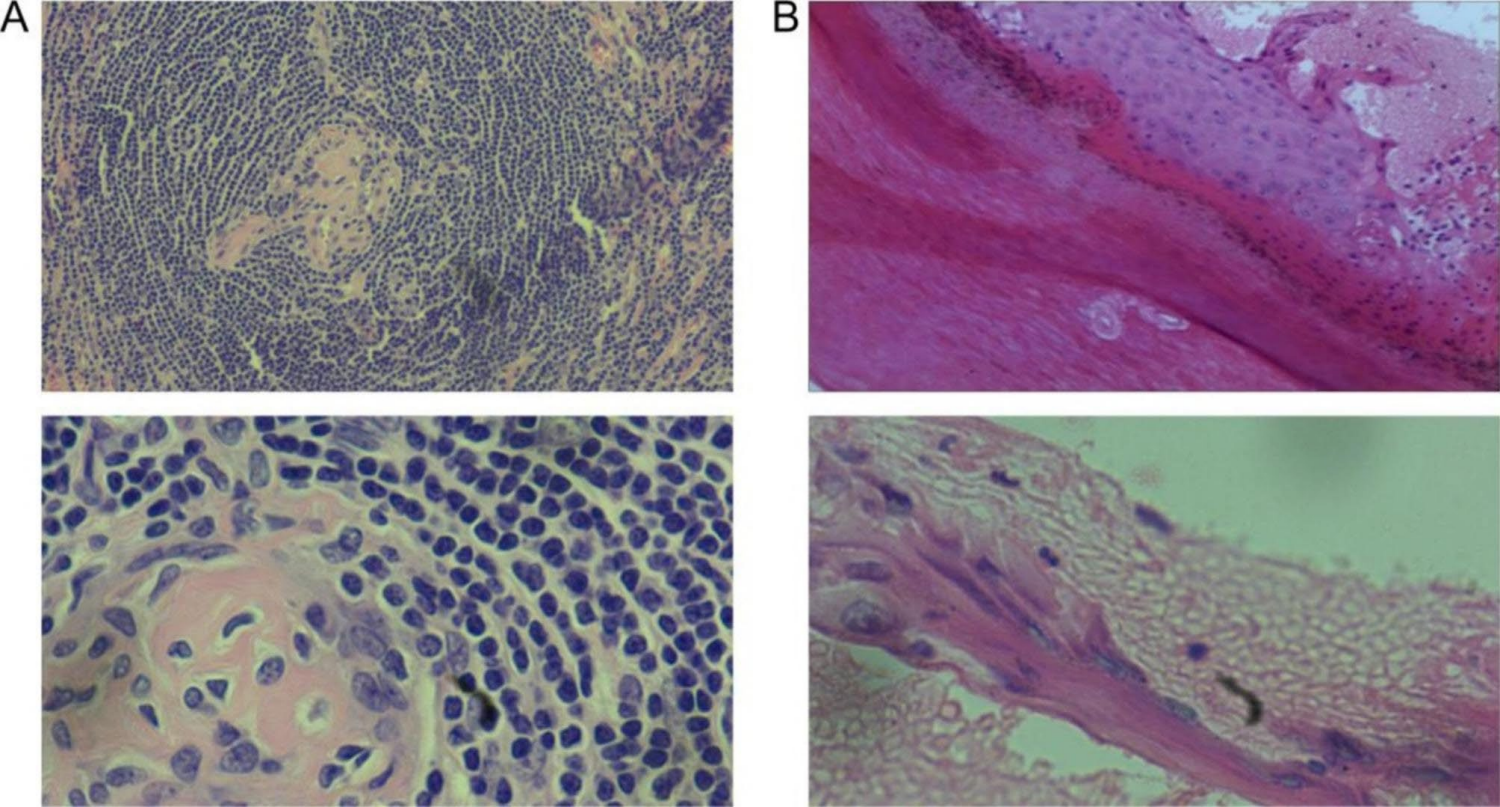
Pathological examination of mass and heel mucosa. (**A**) Pathological image of mass. (**B**) Pathological image of heel mucosa

The mass was biopsied. The pathological examination revealed the classical manifestations of CD (Fig. 2A). Therefore, according to the clinical manifestations and pathological results, the primary diagnosis was SLE and CD. The mucosal skin lesions were considered as lupus vasculitis. The patient was treated with prednisone acetate 1 mg/kg/d, hydroxychloroquine sulfate 100 mg bid, leflunomide 10 mg qd, and thalidomide 25 mg qd. The skin lesions were treated with regularly changed dressing, recombinant human epidermal growth factor spray, sulfadiazine silver cream, mupirocin ointment, red light therapy, and supportive treatments.

**Fig. 3:**
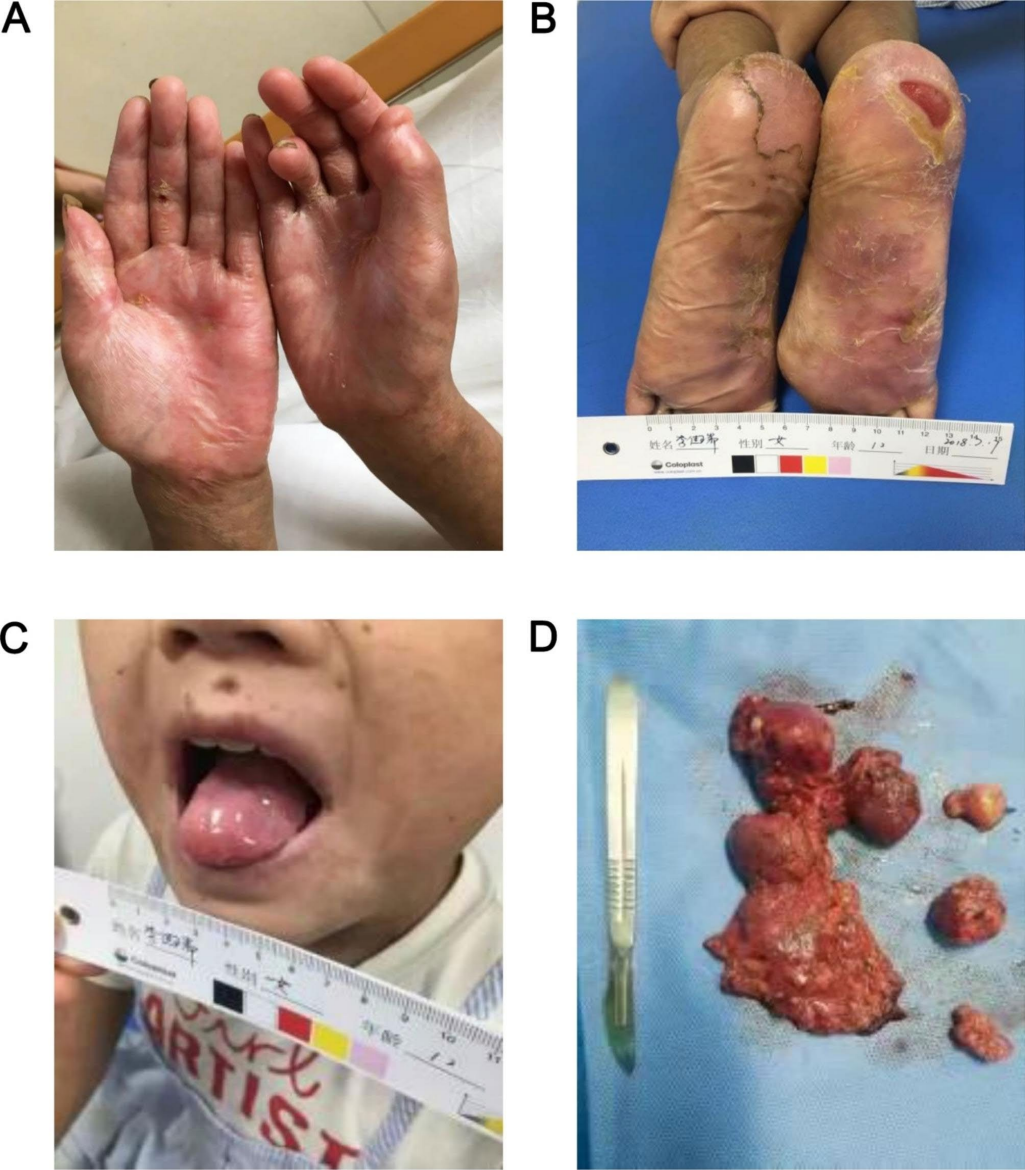
The condition of the children improved after treatments and enlarged lymph nodes in the neck. (**A**) Skin lesions on the palm. (**B**) Skin lesions under the feet. (**C**) The oral mucosa did not improve. (**D**) Specimen of the Castleman disease lesion

In October 2016, the child’s temperature was normal, the skin lesions in the palm were cured (Fig. 3A), the exudations of both feet were reduced (Fig. 3B), and the skin lesions were improved. The blood routine examination was normal. Pericardial effusion disappeared. However,after half a year of the medication treatment, the oral mucosa had not improved (Fig. 3C) until May 2017. The heel skin was used for pathological examination (Fig. 2B). The results showed hyperkeratosis, focal edema, acantholysis of the prickle cell layer, separation at the junction of epidermis and dermis, and blisters that contained red blood cells, lymphocytes, and small neutrophils. Therefore, according to the clinical manifestations and pathological examination, it was considered as PNP.

**Fig. 4:**
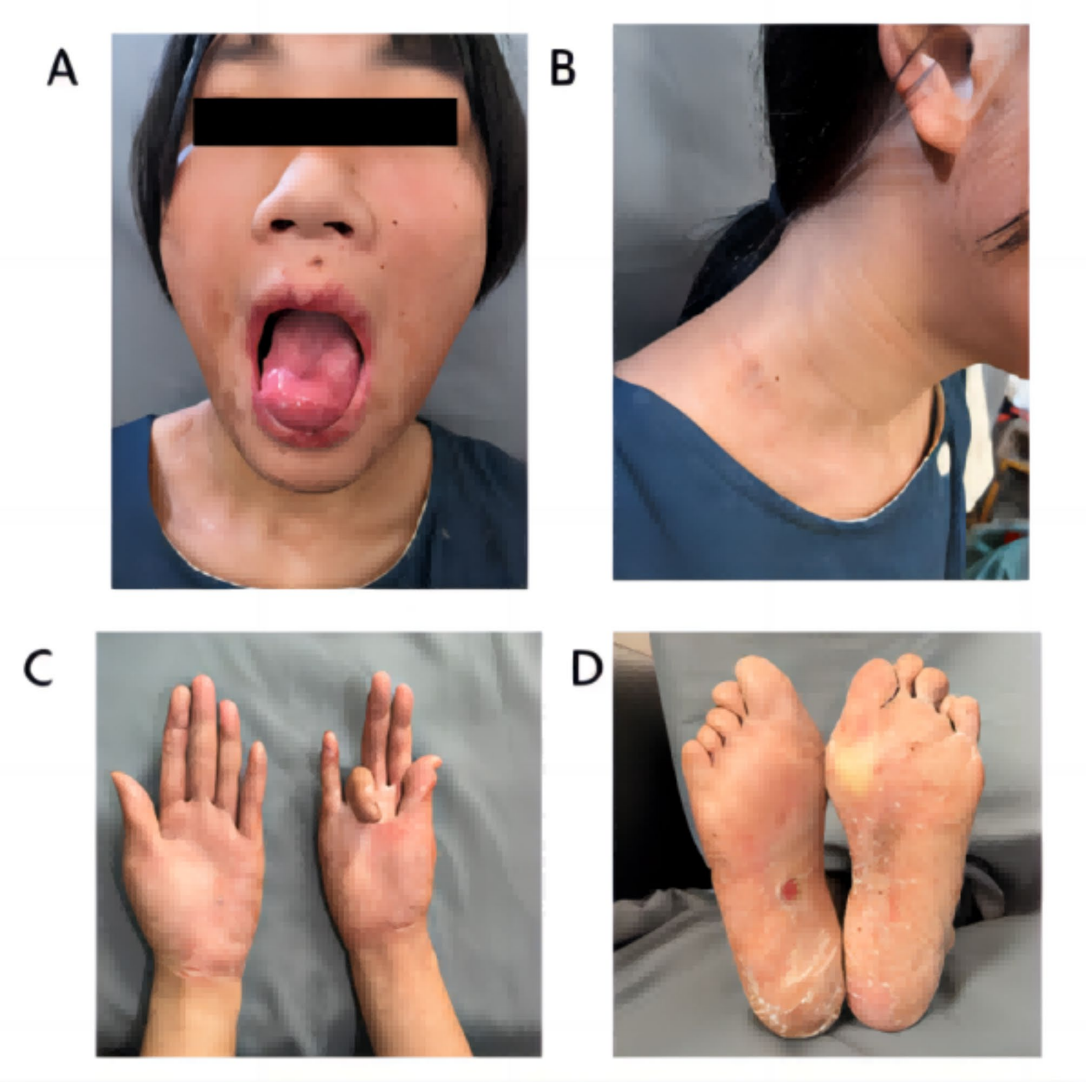
The symptoms improved 6 years after operation, and there is no recurrence. (**A**) The oral mucosa had healed. (**B**) The neck did not appear any new lump. (**C**) Skin lesions on the palm had healed. (**D**) Skin lesions under the feet

It was recommended to remove the tumor (Fig. 3D) and to treat with Tocilizumab (an antagonist of the Interleukin-6 receptor), but her parents refused to use Tocilizumab for treatment due to economic reasons. Therefore, patients were given surgical resection of the enlarged lymph node lesion of the neck. Symptoms associated with PNP were improved and the lump was confirmed as CD by pathological examination. The patient continued to receive oral anti-rheumatic drugs (Prednisone, Leflunomide and Hydroxychloroquine Sulfate) and was followed-up for 3 months. The patient’s limb lesions and oral ulcers have improved significantly, and they have basically healed. Currently the patient has gone to school normally. In 2019, the patient discontinued prednisone and used leflunomide intermittently, and she was not reviewed due to poor compliance. She was followed up in July 2023 with a height of 159 cm and weight of 42.4 kg, and had her first period at the age of 14. Her oral ulcer had healed (Fig. 4A), no new swollen lymph nodes were found at the neck lymph node resection site (Fig. 4B), the skin of her hands was no longer ulcerated (Fig. 4C), but there was a little skin damage on her right foot (Fig. 4D). The results of blood routine urine routine examination were normal at the time of this follow-up, and the complement was normal ,with C3 1.17 g/L and C4 2.5 g/L, while the ANA, anti-ds-DNA,, anti-nucleosome were still positive and IgG was still elevated (18.09 g/L).

## Discussion and conclusions

CD can be misdiagnosed as SLE [19, 20]. Unicentric CD can be associated with SLE [15, 16], but the co-occurrence rate of SLE and CD is probably underreported since lymphadenopathy is a common occurrence during the course of SLE and those enlarged lymph nodes are usually not systematically examined [23]. Demirkan FG introduced the case of a 16-year-old girl with SLE, who had abdominal lymph node enlargement detected by ultrasound and abdominal magnetic resonance imaging, and the histopathological examination of lymph nodes was consistent with CD [19]. PNP can be associated with CD [24]. A study revealed that PNP is relatively common in Chinese patients with CD [25]. Rare cases of PNP combined with SLE have been reported [21, 22]. The rare patient reported here presented SLE, CD, and PNP at the same time. One patient with a past history of SLE and CD was ultimately diagnosed with PNP [21]. Apart from the fact that all three diseases are autoimmunologically mediated, the exact mechanisms responsible for the simultaneous development of all three diseases are unknown, and the rarity of the condition precludes in-depth analyses.

Since all three conditions involve autoimmunity [1–5], the patient reported here was treated with anti-SLE therapies with success. Only the mucosal injury persisted after treatment. It is speculated that oral erosion and injury of hand and foot mucosa are caused by PNP. Finally, the mucosal lesions improved after the CD mass was removed, which also confirmed that these skin and mucosal lesions were caused by PNP. She is still alive at 5 years after PNP diagnosis (the actual course of the disease was, in fact, longer) and the reported 1-year survival rate is only 25% in patients with PNP but can be 90% with treatments [10, 13].

Siltuximab is a monoclonal antibody targeting IL-6. As the only drug approved by the US Food and Drug Administration (FDA) and the European Drug Administration for the treatment of multicentric CD (MCD), it is recommended as a first-line drug in the treatment guidelines [26]. However, there are few reports of cases of CD complicated with PNP treated with siltuximab. Tocilizumab is currently approved in Japan for treating MCD. The use of tocilizumab (8 mg/kg every 2 weeks) may also be effective in the absence of siltuximab [9].

Unicentric CD (UCD) is limited and has a good prognosis, and surgical resection is still the best choice [27]. A previous case report of a patient with SLE combined with CD and PNP suggested that the removal of the benign neoplasm associated with CD could lead to the complete remission of PNP [21]. Castleman Disease Collaborative Network (CDCN) recommends a complete surgical resection as the preferred intervention for UCD whenever possible, and UCD recurrence after complete surgical resection is rare [27]. Patients with UCD and PNP could be benefit from complete surgical removal of UCD, which often halts or reverses the PNP [28]. The girl was developed UCD with PNP and she was followed without recurrence for 6 years after surgery, which also confirmed the above view. CDCN recommends that these patients need continuous follow-up for relevant clinical manifestations following complete surgical management. Moreover, these patients should be carefully evaluated to avoid misdiagnosis of other diseases that may cause related manifestations, such as iMCD, autoimmune disease, endocrine disorders, or fibromyalgia. Appropriate testing (e.g., antinuclear antibodies, ESR, CRP, cytokine combinations) can assist physician to identify the presence of other inflammatory diseases [27]. At the final visit, the girl remained positive for ANA and dsDNA antibodies. We will continue to follow her laboratory tests in the future.

In summary, we report a rare case of concurrent SLE, CD and PNP in a 12-year-old female patient. With insufficient improvement, especially of the mucosal changes under immunosuppressive therapy, healing occurred only after removal of the Castleman tumour, highlighting its pathophysiological significance for the skin changes and PNP, respectively, and illustrating that reassessment of the diagnoses is crucial for effective treatment.

## Data Availability

The datasets used and/or analysed during the current study are available from the corresponding author on reasonable request.

## Abbreviations

SLE: Systemic lupus erythematosus
CD: Castleman’s disease
PNP: paraneoplastic pemphigus
IL-6: Interleukin-6
ANA: antinuclear antibody
dsDNA: Anti-double-stranded DNA antibody
CDCN: Castleman Disease Collaborative Network
